# Supplementary material for: Quantitative intra-Golgi transport and organization data suggest the stable compartment nature of the Golgi
Source: eLife. 2025 Jul 8;13:RP98582. doi: 10.7554/eLife.98582 (PMC12237403; doi:10.7554/eLife.98582)
Supplement: Figure 2—source data 1. — n, the number of quantified cells. SEM, standard error of the mean. [file elife-98582-fig2-data1.pdf]

Figure 2-Source Data 1

A. SBP-TNFa-GFP

Chase time (min)	*n*	*LQ*	SEM	Cell number
5	89	-0.18	0.04	12
10	77	0.29	0.05	15
20	54	1.03	0.09	15
40	45	0.77	0.17	30
60	76	1.10	0.13	38
120	54	0.90	0.19	29
180	42	1.25	0.21	27

B. SBP-GFP-CD8a-furin-Y+AC #1

10	38	0.10	0.04	9
20	60	0.65	0.06	9
30	32	0.74	0.05	7
40	42	0.82	0.07	14
60	21	0.89	0.14	8
70	10	0.73	0.16	9

C. SBP-GFP-Tac

10	58	0.04	0.02	4
20	87	0.57	0.03	3
30	82	0.86	0.04	6
40	55	0.80	0.04	2
60	70	0.90	0.04	3
90	50	0.82	0.03	2

D. SBP-GFP-Ecadherin #1

5	52	0.03087	0.02753	11
10	55	0.38	0.03	15
20	31	0.74	0.06	14
40	29	0.85	0.12	11
60	22	0.89	0.15	11
70	18	1.07	0.10	11

E. SBP-GFP-CD8a-furin-YA #2

10	13	0.08	0.05	11
20	16	0.86	0.12	10
40	27	1.29	0.06	16
60	48	1.36	0.06	11
120	58	1.43	0.04	6
240	39	1.37	0.08	11
360	38	1.51	0.05	10
480	76	1.85	0.07	5

F. SBP-GFP-CD59

5	84	-0.01	0.02	12
10	119	0.15	0.02	19
20	131	0.60	0.03	16
30	90	0.85	0.04	18
40	51	0.80	0.05	10
50	87	0.90	0.04	15
60	49	0.96	0.07	15
70	52	0.95	0.06	12

10	150	0.24	0.03	14
15	283	0.43	0.02	14

G. SBP-GFP-Tac-TC
293T

20	147	0.59	0.03	11
40	94	0.76	0.03	8
60	208	0.84	0.02	13
120	93	0.97	0.03	10
180	88	0.99	0.02	9
240	86	0.94	0.02	10

H. SBP-GFP-Tac-TC

10	64	0.02	0.03	2
20	41	0.33	0.04	5
30	74	0.57	0.03	3
40	87	0.80	0.02	5
60	97	0.93	0.02	6
90	130	1.08	0.03	5
120	86	1.10	0.03	2
180	91	1.09	0.04	4
300	80	0.99	0.02	2

I. SBP-GFP-CD8a-furin-WT #1

10	17	-0.03	0.04	14
20	49	0.16	0.05	16
40	33	1.13	0.08	14
60	45	1.31	0.08	10
120	42	1.53	0.07	10
240	34	1.51	0.11	17
360	63	1.60	0.09	18
480	63	1.66	0.08	16

J. SBP-GFP-CD8a-furin-AC #1

20	48	0.92	0.06	11
40	28	1.38	0.10	23
60	26	1.53	0.09	21
120	12	1.71	0.10	21
150	13	1.75	0.15	11
