# Supplementary material for: Quantitative intra-Golgi transport and organization data suggest the stable compartment nature of the Golgi
Source: eLife. 2025 Jul 8;13:RP98582. doi: 10.7554/eLife.98582 (PMC12237403; doi:10.7554/eLife.98582)
Supplement: Figure 2—figure supplement 1—source data 1. — n, the number of quantified cells. SEM, standard error of the mean. [file elife-98582-fig2-figsupp1-data1.pdf]

Figure 2-Figure Supplement 1-Source Data 1

A. SBP-GFP-CD59
293T#1

Chase time (min)	*n*	*LQ*	SEM	Cell number
10	85	0.29	0.04	10
20	45	0.69	0.05	7
30	58	0.61	0.05	5
40	52	0.67	0.05	7
60	24	0.64	0.10	5

B. SBP-GFP-CD59
293T#2

5	64	0.12	0.03	15
10	27	0.28	0.09	8
20	97	0.48	0.04	16
30	54	0.67	0.06	10
40	24	0.62	0.10	11
60	15	0.51	0.09	12

C. SBP-GFP-CD59
293T#3

5	244	0.43	0.02	13
10	222	0.48	0.03	12
15	232	0.68	0.02	14
20	212	0.70	0.03	15
30	307	0.82	0.02	16
40	115	0.76	0.03	8
60	126	0.71	0.03	16
80	94	0.81	0.04	9

D.TfR-SBP-GFP

5	76	0.16	0.02	10
10	95	0.51	0.03	8
15	83	0.82	0.03	11
20	58	0.95	0.06	10
30	37	1.10	0.08	10
40	37	1.10	0.05	13

E. SBP-GFP-CD8a-furin-Y+AC #2

10	67	0.15	0.04	13
20	62	0.69	0.06	11
30	27	0.68	0.07	13
40	39	0.89	0.09	14
60	28	1.00	0.08	15
70	16	1.04	0.09	13

F. SBP-GFP-CD8a-furin-Y+AC #3

10	44	0.31	0.03	9
20	82	0.62	0.04	12
25	89	0.95	0.04	11
30	72	0.94	0.04	15
40	40	0.99	0.05	14
50	49	0.97	0.05	14

G. SBP-GFP-Tac #2

5	59	-0.02	0.06	12
7	77	0.05	0.04	12
10	78	0.23	0.05	13
15	127	0.54	0.03	15
20	127	0.58	0.04	13
25	132	0.70	0.04	14

30	97	0.82	0.04	10
50	99	0.91	0.04	13
80	96	0.89	0.05	12

H. SBP-GFP-Ecadherin #2

5	54	0.02	0.04	12
10	55	0.31	0.04	16
20	103	0.91	0.05	15
40	44	1.06	0.05	13
60	36	1.03	0.17	13
70	34	1.07	0.24	13

I. SBP-GFP-Ecadherin #3

5	43	0.18	0.03	10
10	43	0.52	0.06	12
20	39	1.00	0.07	8
40	36	1.01	0.09	16
60	11	1.14	0.23	9
70	21	1.14	0.12	13

J. SBP-GFP

10	67	0.02	0.04	6
15	84	0.19	0.04	9
20	124	0.33	0.02	6
30	138	0.55	0.03	10
40	207	0.67	0.02	10
50	105	0.64	0.03	8
80	82	0.71	0.03	9

K. SBP-GFP-CD8a-furin-YA #2

10	32	0.07	0.04	5
20	28	1.00	0.10	6
40	59	1.51	0.07	12
60	65	1.61	0.07	11
120	38	1.48	0.05	6
240	100	1.54	0.04	11
360	73	1.39	0.04	11
480	86	1.50	0.04	5

L. SBP-GFP-CD8a-furin-YA #3

10	20	0.25	0.04	15
20	15	0.63	0.12	14
40	35	1.27	0.08	13
60	43	1.22	0.07	10
120	46	1.28	0.08	6
240	44	1.35	0.09	16
360	64	1.52	0.05	8
480	48	1.67	0.07	6

M. SBP-GFP-Tac-TC
293T#2

10	128	0.32	0.03	15
20	237	0.63	0.02	13
40	193	0.82	0.02	13
60	100	0.94	0.03	7
120	58	1.02	0.03	10
180	62	0.97	0.04	15
240	102	0.99	0.03	13

N. SBP-GFP-Tac-TC 293T#3

20	83	0.45	0.04	24
40	43	0.81	0.03	15
60	24	0.78	0.07	8
90	25	0.96	0.04	7
120	18	0.88	0.06	8
180	16	1.08	0.05	4
240	21	1.01	0.07	11

O. SBP-GFP-Tac-TC #2

5	21	-0.18	0.17	11
7	16	-0.23	0.17	9
10	36	0.19	0.07	12
15	70	0.40	0.07	9
25	119	0.69	0.09	11
30	127	0.77	0.03	10
40	126	0.84	0.03	10
50	178	0.93	0.03	11
80	112	0.99	0.02	9
110	121	1.11	0.03	9
140	113	1.18	0.03	12
200	182	1.19	0.02	11

P. SBP-GFP-CD8a-furin-WT #2

10	48	0.04	0.02	8
20	25	0.28	0.07	9
40	40	1.28	0.08	10
60	49	1.34	0.07	15
120	46	1.37	0.08	13
240	31	1.47	0.13	11
360	55	1.55	0.06	14
480	45	1.79	0.09	10

Q. SBP-GFP-CD8a-furin-WT #3

5	89	0.02	0.02	9
10	89	0.27	0.02	10
20	100	0.64	0.05	8
30	116	1.32	0.04	18
40	131	1.42	0.03	9
60	127	1.23	0.06	17
90	90	1.51	0.05	9
120	82	1.47	0.05	17
150	105	1.57	0.05	13
180	108	1.56	0.05	13

R. SBP-GFP-CD8a-furin-AC #2

20	30	0.67	0.06	9
40	28	1.01	0.09	8
60	25	1.21	0.08	7
120	14	1.42	0.17	9
150	12	1.50	0.21	7

S. SBP-GFP-CD8a-furin-AC #3

20	35	0.80	0.07	10
40	14	1.06	0.17	10
60	24	1.39	0.16	15
120	11	1.62	0.14	8
150	12	1.52	0.10	11

T. SBP-GFP-CD8a-furin-AC #4

10	22	0.15	0.04	12
20	29	0.67	0.06	15
40	17	1.17	0.14	18
60	32	1.40	0.08	19
90	23	1.60	0.16	17
120	23	1.69	0.18	17
150	20	1.44	0.21	11
240	13	1.51	0.14	10
480	19	1.73	0.11	8
