# Supplementary material for: Quantitative intra-Golgi transport and organization data suggest the stable compartment nature of the Golgi
Source: eLife. 2025 Jul 8;13:RP98582. doi: 10.7554/eLife.98582 (PMC12237403; doi:10.7554/eLife.98582)
Supplement: Figure 3—source data 1. — n, the number of quantified cells. SEM, standard error of the mean. [file elife-98582-fig3-data1.pdf]

Figure 3-Source Data 1

A. SBP-GFP-Collagen X #1

Chase time (min)	*n*	*LQ*	SEM	Cell number
5	143	0.23	0.04	11
10	263	0.44	0.03	17
15	201	0.57	0.04	11
20	150	0.85	0.04	12
30	96	0.91	0.05	9
40	69	0.91	0.05	10
60	90	0.99	0.08	13
90	44	0.95	0.15	11

B. SBP-GFP-Collagen X #2

5	211	0.04	0.03	11
10	229	0.32	0.03	11
15	175	0.41	0.03	8
20	158	0.59	0.05	9
30	143	0.80	0.05	15
40	235	0.77	0.05	15
60	87	0.80	0.09	16
90	53	0.78	0.13	12

C. SBP-GFP-Collagen X #3

5	196	0.06	0.03	8
10	286	0.36	0.03	15
15	167	0.68	0.04	9
20	272	0.69	0.04	14
40	111	0.90	0.06	13
60	92	0.88	0.08	13
90	43	0.84	0.16	13

E. SBP-GFP-Collagen X side-averaging

Chase time (min)	*n*	LQ^{side}	SEM	Cell number
5	31	0.11	0.07	10
10	48	0.33	0.06	10
20	37	0.86	0.08	8
40	34	0.97	0.10	8
