# Supplementary material for: Quantitative intra-Golgi transport and organization data suggest the stable compartment nature of the Golgi
Source: eLife. 2025 Jul 8;13:RP98582. doi: 10.7554/eLife.98582 (PMC12237403; doi:10.7554/eLife.98582)
Supplement: Figure 6—source data 1. — n, the number of quantified cells. SEM, standard error of the mean. [file elife-98582-fig6-data1.pdf]

Figure 6-Source Data 1

	BFA treatment time(min)	*n*	*LQ*	SEM	Cell number
TGN46	0	102	1.56	0.04	5
	10	97	1.47	0.08	18
	20	68	1.30	0.05	13
	30	62	1.25	0.05	19
ST6GAL1-moxGFP	0	89	0.90	0.03	8
	10	116	0.95	0.02	7
	20	133	0.97	0.02	17
	30	91	0.93	0.03	12
GS15	0	110	0.70	0.03	9
	10	89	0.68	0.03	10
	20	28	0.74	0.07	11
	60	20	0.65	0.08	12
GFP-Golgin-84	0	78	0.05	0.05	8
	10	33	0.04	0.08	15
	20	52	0.10	0.15	14
	30	48	0.40	0.11	13
CD8a-furin	0	70	2.22	0.12	9
	10	59	1.73	0.17	18
	20	34	1.51	0.24	16
	30	41	1.39	0.15	19
CD8a-CI-M6PR	0	47	2.37	0.21	15
	10	29	1.77	0.21	19
	20	32	1.63	0.14	12
	30	36	1.29	0.18	19

	BFA treatment time(min)	*n*	*d*	SEM	Cell number
$d_{(GM130\text{-}GalTmCherry)}$ side-averaging	0	34	300	10	9
	10	18	280	27	11
	20	28	160	20	15
	30	13	190	32	7

	BFA treatment time(min)	*n*	*d*	SEM	Cell number
$d_{(GalTmCherry\text{-}CD8afurin)}$ side-averaging	0	30	210	21	7
	10	21	180	28	10
	20	21	90	28	9
	30	20	100	28	10
